# Perspective: Challenges in Use of Adolescent Anthropometry for Understanding the Burden of Malnutrition

**DOI:** 10.1093/advances/nmy133

**Published:** 2019-05-02

**Authors:** Alison Tumilowicz, Ty Beal, Lynnette M Neufeld, Edward A Frongillo

**Affiliations:** 1 Global Alliance for Improved Nutrition (GAIN), Geneva, Switzerland; 2 Department of Environmental Science and Policy, University of California, Davis, CA; 3 Center for Research in Nutrition and Health Disparities, Arnold School of Public Health, University of South Carolina, Columbia, SC

**Keywords:** adolescence, maturation, puberty, peak height velocity, anthropometry, growth references, height-for-age *z* score, body mass index

## Abstract

Improving nutritional status during adolescence is an opportunity to improve the lives of this generation and the next. Estimating the burden of malnutrition at a population level is fundamental to targeting interventions and measuring progress over time, and for adolescents, we usually depend on survey data and the 2007 WHO Growth Reference to do so. There is substantial risk of misguided conclusions regarding adolescent prevalence estimates, however, when underlying methodological limitations of the indicators and reference are not adequately considered. We use national prevalence estimates among girls and young women 10–22 y of age from the 2014 State of Food Security and Nutrition in Bangladesh report as an example to demonstrate that determining the true prevalence of undernutrition, overweight, and obesity is complicated by racial/ethnic variation across populations in timing of the adolescent growth spurt, growth potential, and body build. Further challenging the task are inherent limitations of the body mass index as an indicator of thinness and adiposity, and cutoffs that poorly distinguish a well-nourished population from a malnourished one. We provide recommendations for adolescent nutrition policy and program decision-making, emphasizing the importance of *1*) critically interpreting indicators and distributions by age when using the 2007 WHO Growth Reference; *2*) examining what is happening before and after adolescence, when interpretation of anthropometry is more straightforward, as well as trends over time; and *3*) complementing anthropometry with other information, particularly dietary intake. Finally, we advocate that nutrition researchers prioritize exploration of better methods to predict peak height velocity, for development of standardized indicators to measure dietary quality among adolescents, and for studies that will illuminate causal paths so that we can effectively improve adolescent dietary intake and nutritional status.

## Introduction

Adolescent nutrition is coming of age, gaining significant attention in the past few years (1, 2). The recent surge in population growth makes this the largest generation of adolescents in history, most of whom live in low- and middle-income countries (3). Although the first 1000 d remains a critical period of physical growth and nutritional need, requiring continued high global attention, there is good reason to also focus on adolescence—generally defined as individuals aged 10–19 y (4, 5). During the adolescent growth spurt or period of peak height velocity, the growth rate for girls is similar to—and for boys surpasses—the rate at 2 y of age (6). Adolescence is the only time in life besides early infancy when the velocity of growth increases (7). During puberty, adolescents gain ∼15% of their final adult height. By about age 20 y, 90–95% of total peak bone mass is attained, 45% of which is built during adolescence (7–9). Weight gain during this period accounts for about half of the ideal adult weight. Rapid biological and psychosocial growth and development increase nutritional needs for both boys and girls (10). Finally, recent evidence has reopened the question of whether interventions during adolescence could redress linear growth deficits accumulated earlier in life (11, 12).

Interest in adolescent nutrition has generated evidence reviews (13–16), new global guidelines (17), international meetings (18–20), donor commitments, and political will to institute new policies and programs (21, 22). To effectively channel these efforts and resources, precise and context-specific evidence is required for decision-making regarding which adolescent nutrition interventions should be implemented for particular purposes and populations (23). Anthropometric indicator prevalence estimates based on cross-sectional survey data and the 2007 WHO Growth Reference for children and adolescents 5–19 y old (24) (hereinafter referred to as the 2007 WHO Growth Reference) are frequently used to assess and compare the burden of malnutrition at a population level, including comparisons over time and across age subgroups. Several such analyses have been recently conducted and reviews written using large-scale cross-sectional survey data such as the Demographic and Health Surveys and Global School-Based Student Health Surveys (15, 16, 25–27). Although some of these articles include caveats related to the robustness of prevalence estimates, the continued use of these data, references, and cutoffs holds substantial risk for misguided conclusions being reached related to the magnitude and distribution of nutritional issues in populations.

**FIGURE 1:**
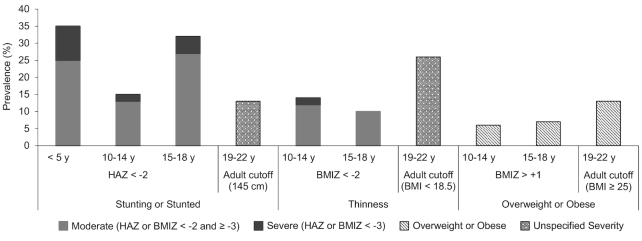
Prevalence of stunting, thinness, and overweight or obesity in girls and women by age and cutoff in Bangladesh. Severe stunting is defined as a height-for-age *z* score <−3 SD and moderate stunting as <−2 SD but ≥−3 SD from the 2006 WHO Child Growth Standard for children <5 y (29) and from the 2007 WHO Growth Reference for children and adolescents 5–19 y (24). Overweight or obesity in girls 10–18 y is defined as a BMIZ > +1 SD from the 2007 WHO Growth Reference for children and adolescents 5–19 y. Source: Reference (28). BMI in kg/m^2^. BMIZ, BMI-for-age *z* score; HAZ, height-for-age *z* score.

We use national prevalence estimates of stunting, thinness, and overweight or obesity in girls 10–22 y old from the 2014 State of Food Security and Nutrition in Bangladesh (SFSNB) report to illustrate the challenge (**Figure 1**) (28). When taking the indicators at face value, it appears that stunting is high in girls aged <5 y, drops 2-fold in girls 10–14 y old, increases 2-fold in girls 15–18 y old, and drops >2-fold in women 19–22 y old. Moreover, the prevalence of thinness more than doubles among girls 19–22 y old compared with 15–18 y old. Such dramatic fluctuations in linear growth and weight status are biologically improbable and, as we will explain, are, in part, a result of underlying methodological limitations of anthropometric indicators during adolescence and the 2007 WHO Growth Reference.

We bring to the fore and update, based on current evidence, fundamental challenges to assessing adolescent growth using cross-sectional data. We aim to bring clarity to the interpretation of prevalence estimates based on the 2007 WHO Growth Reference for policy and program decision-making. We briefly describe the 2007 WHO Growth Reference and outline 6 issues that influence estimates or the meaning of adolescent anthropometric indicators: *1*) adjustment for timing of the adolescent growth spurt, *2*) racial/ethnic differences in linear growth potential, *3*) limitations of BMI (in kg/m^2^) as an indicator of adiposity and thinness among adolescents and across different racial/ethnic populations, *4*) changes in the SD of the denominator used for calculating height-for-age *z* scores (HAZ) and BMI-for-age *z* scores (BMIZ), *5*) the relation between cutoffs and risk of adverse outcomes, and *6*) alignment of cutoffs across the lifespan. We conclude with a discussion of what can be done now and in the long term to support estimates of the magnitude and distribution of nutrition issues in adolescent populations and evidence-informed decisions about the types of adolescent nutrition interventions needed to address them.

## The 2007 WHO Growth Reference

### Standard compared with reference

In 1995, the WHO Expert Committee on Physical Status: The Use of and Interpretation of Anthropometry defined a reference as “a tool for grouping and analyzing data [that] provides a common basis for comparing populations; no inferences should be made about the meaning of observed differences” (30). This contrasts with a standard, which “embraces the notion of a norm or desirable target, and thus involves a value judgement” (30). Operationally, a reference describes the growth pattern of a specific population, whereas a standard defines a recommended growth pattern that has been associated with specified health outcomes (31). A growth reference is developed descriptively (32). Typically a nationally representative distribution is constructed using mild exclusion criteria to select a supposed healthy, well-nourished population, but the observed distribution is susceptible to being composed of 2 sub-distributions—a healthy one and an unhealthy one (30, 32). Consequently, a reference could be skewed to one tail or the other depending on the position of the unhealthy population distribution, having a larger variance than if unhealthy individuals were excluded. In contrast to a growth reference, a growth standard is developed prescriptively (33). The 2006 WHO Child Growth Standard for children from birth to 5 y (29) was constructed using longitudinal and cross-sectional data collected prospectively through the Multicentre Growth Reference Study (MGRS) (34). Children from the 6 participating countries (Brazil, Ghana, India, Norway, Oman, and the United States) were healthy, fed according to WHO feeding recommendations, and met other rigorous criteria to ensure there were no significant environmental or individual constraints on growth (34). The MGRS found striking similarity in linear growth between children in the 6 sites, justifying pooling the data and constructing a single international standard from birth to 5 y of age (35). As a result, the 2006 WHO Child Growth Standard reflects how children should grow under optimal conditions and can be used to make inferences about health or nutrition (33).

### Development of the 2007 WHO Growth Reference

In 2003, an expert meeting brought together representatives from WHO, the United Nations University Food and Nutrition Program, and the FAO to consider the feasibility and appropriateness of developing a single international growth reference or standard to describe universal growth patterns of children and adolescents aged 5–19 y (31). The expert group concluded that it could not be ruled out that some differences in linear growth across populations reflect genetics rather than solely environmental factors (36). Therefore, the sampling frame for the development of an international growth standard for children >5 y old would have to include a multiethnic sample to capture the variation in human growth patterns. The expert group also determined that a prescriptive standard using either historical or prospective growth data is possible with careful consideration of the population and individual selection criteria, study protocol, and statistical methods. Nevertheless, the expert group decided it would not be feasible to conduct a prospective, prescriptive, multicenter study, like the MRGS, owing to the difficulty of controlling the dynamics of older children's environments. Turning attention to existing data sets, they found excessive heterogeneity in study designs, socioeconomic status of participating children, and other factors critical to growth curve construction. Subsequently, the expert group developed the 2007 WHO Growth Reference by reconstructing the 1977 United States National Center for Health Statistics (NCHS)/WHO Growth Reference which was based on US children collected by the US Health Examination Surveys from 1960 to 1975 (37).

**FIGURE 2:**
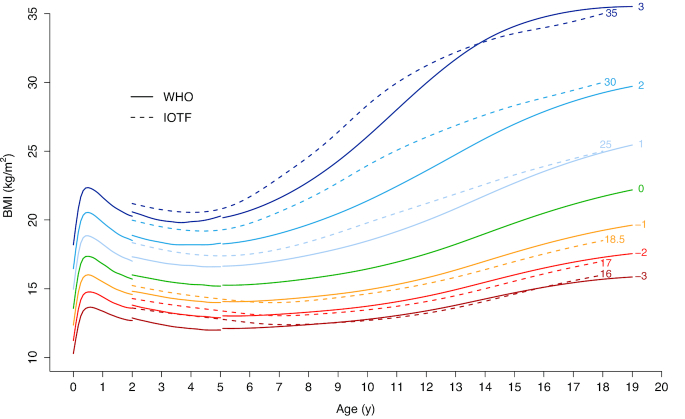
WHO and IOTF BMI growth curves for boys. WHO growth curves for children <5 y are based on the 2006 WHO Child Growth Standard (29). WHO growth curves for children and adolescents 5–19 y are based on the 2007 WHO Growth Reference (24). IOTF curves are based on the 2012 IOTF extended cutoffs for children and adolescents 2–18 y (40). Data were fitted using cubic smoothing splines. Numbers at the end of the IOTF growth curves (dotted lines) specify the corresponding adult BMI values. Numbers at the end of the WHO curves represent the corresponding *z* scores (SDs). Colors are used to differentiate between different levels of severity. Severity levels are defined as follows: BMI < 16 or BMIZ < −3, grade 3 (severe) thinness; BMI < 17 or BMIZ < −2, grade 2 (moderate) thinness; BMI < 18.5 or BMIZ < −1, grade 1 (mild) thinness; BMI ≥ 25 or BMIZ ≥ +1, overweight; BMI ≥ 30 or BMIZ ≥ +2, obesity; BMI ≥ 35 or BMIZ ≥ +3, severe obesity. For children aged 0–5 y, WHO defines overweight as BMIZ ≥ +2 and obesity as BMIZ ≥ +3. BMIZ, BMI-for-age *z* score; IOTF, International Obesity Task Force.

The expert group recognized the inherent limitation of using data based on a single population, which does not account for genetic differences in growth potential (36, 38). Nevertheless, the 2007 WHO Growth Reference is an improvement on the NCHS/WHO Growth Reference in that it includes BMI and the curves align with the 2006 WHO Child Growth Standards at 5 y and recommended adult cutoffs for overweight and obesity. Moreover, the reference population of US children from 1960 to 1975 came from mostly nondeprived circumstances and were yet to be greatly affected by the obesity epidemic. If the reference population were undernourished, the “true” prevalence of a specified indicator of poor nutrition (e.g., severe stunting or severe thinness) for the study population would be underestimated. Inversely, using a reference with a positively skewed BMI distribution to assess children worldwide would result in an underestimation of the prevalence of child overweight and obesity and overestimation of undernutrition. For example, the International Obesity Task Force (IOTF) reference for children aged 2–18 y uses data from 6 different countries and is thus more internationally representative (39, 40), but the IOTF reference is based on more recent data. Using the IOTF reference, compared to the 2007 WHO Growth Reference, results in overall higher estimates of thinness and lower estimates of overweight and obesity (40, 41) (**Figure 2**).

## Issues Affecting Prevalence Estimates for Adolescent Anthropometric Indicators Using the 2007 WHO Growth Reference

### Population estimates need to be adjusted for timing of the adolescent growth spurt

Population estimates need to be adjusted for timing of the adolescent growth spurt. Growth occurs in tandem with pubertal development. There is large individual and population variability in the age that biological maturation begins and reaches completion, depending on genetic and environmental factors (42). Different population groups may mature at different times, velocities, and intensities. For example, the median age of menarche can vary from 12 to 18 y (43, 44) and has reduced in some populations as social and economic conditions have improved by as much as 1 y per decade over the span of 25 y (45). Secular changes in the onset of the adolescent growth spurt or period of peak height velocity have also been documented (46, 47). Because of this variability, age is a poor benchmark of biological maturation and nutritional needs (6, 30). The reference population may mature at a different time, velocity, and intensity than the population under study. Adjusting only for age and sex in growth curves poorly calibrates for the period of peak height velocity and, therefore, height-for-age and BMI-for-age indicators can grossly misrepresent nutritional status (30). A study population that matures at a later chronological age than the reference population may appear to be experiencing growth faltering, whereas in reality they are growing adequately but peak height velocity has not started (the inverse happening for earlier-maturing populations). The weight–height relation also dramatically changes with maturation (48).

Weight:height ratios that use a scaling exponent or power for height (*p*), such as a Benn index (weight/height*^p^*), are intended to correct for the correlation between weight and height and thereby provide a measure of body shape independent of height (49, 50). In adults, Quetelet's index, better known as BMI, uses a *p* equal to 2.0, which accounts for most of the correlation between weight and height (50, 51). The weight–height relation depends on age, however, and is largest when weight is growing fastest relative to height, which happens in infancy and adolescence when *p* is ≥3 compared to 1.5 in mid-childhood (38, 48, 52–54). The *p* to which height should be raised to correct for the correlation between weight and height fluctuates during adolescence and depends on sex and maturation (52, 53, 55). During the adolescent growth spurt, adolescents tend to be taller than average and relatively heavy for their height (52, 56, 57). Subsequently, a larger height power is needed during the period of peak height velocity than before or after (48, 52). Adolescents whose attained height is a result of the adolescent growth spurt are heavier than those of the same height who are less mature. If the scaling exponent is less than what is required to correct for the weight–height correlation, then as height increases, the height*^p^* denominator of the index is smaller than it should be. In other words, taller and more mature adolescents will have a higher BMI than they should have and be more likely to be classified as overweight or obese than shorter and less mature adolescents. If the height distributions at all ages in the reference and study populations were similar, then a constant scaling exponent across ages would not affect prevalence estimates of overweight or obesity. Because age is a poor marker of the adolescent growth spurt, however, it remains important to align maturation between the reference and study populations. Therefore, BMI provides a valid estimate of body shape during puberty only if the height distributions at all ages in the reference and study populations are similar and adjustment is made for biological maturation.

Not adjusting for biological maturation can substantially alter conclusions, as was demonstrated in a cross-cultural comparison of 2 adolescent populations in Senegal and Martinique (58). Girls were measured at ∼14 y of age. Girls in Martinique had significantly higher mean ± SD BMI (Martinique: 20.5 ± 4.1 compared with Senegal: 18.0 ± 2.4, *P* < 0.0001) and 19% were classified as overweight or obese, whereas in Senegal there were virtually no overweight or obese adolescents. Girls from Martinique were more sexually mature, however, than adolescent girls from Senegal (94.6% in Martinique and 12.2% in Senegal had experienced menarche). When comparisons were repeated after Senegalese girls reached menarche, differences in mean weight and BMI disappeared.

Based on stages of sexual maturation, originally specified by Tanner (59) (referred to as Tanner stages), the 1995 WHO Expert Committee on Physical Status recommended clinical examination of 2 markers for each sex to align chronological age and occurrence of peak height velocity of reference and sample populations: one signaling onset of the growth spurt in height or “takeoff” (Tanner stage 2 breast stage for girls, Tanner stage 3 genitalia stage for boys) and another indicating that peak height velocity and related changes have completed (menarche for girls, adult voice for boys) (30). The underlying rationale for this method is that linear growth occurs parallel to pubertal development, with the activation of the hypothalamic–pituitary–gonadal axis as the proposed driver of the adolescent growth spurt; median ages at which secondary sex characteristics are expressed coincide with the timing and tempo of peak height velocity (45). Maturational events were not collected from the 1960–1975 NCHS/WHO reference population, the same reference population of the 2007 WHO Growth Reference. Median and mean age of menarche (both 12.8 y) were estimated at the national level from Cycles II and III of the Health Examination Survey, however, which surveyed parents of adolescents (*n* = 2242 girls aged 11–14 y) between 1963 and 1970 (60). Considering median age of menarche can vary by as much as 6 y across different populations, this adjustment could significantly alter prevalence estimates.

In the example of Bangladesh, the 2014 SFSNB survey did not collect information on maturation. A study conducted in 2014 among 680 urban high school girls in the Bongaon area of the Jessore district in southwest Bangladesh, however, found a mean age of menarche of 11.6 y (44). A study conducted in 2005 among 3923 rural girls from the Gaibandha district in northwest Bangladesh, an area with below-average socioeconomic status, found a mean age of menarche of 12.8 y (61). Given that about two-thirds of Bangladesh is rural, it is reasonable to assume the mean age of menarche in the 2014 SFSNB sample of girls was 12.4 y, ∼5 mo earlier than the NCHS/WHO reference population (60). Among Bangladeshi girls aged 10–14 y, prevalence of stunting and thinness could be higher and overweight or obesity lower than they appear in Figure 1.

The Tanner stages were based on a limited sample size of Caucasian children living in the United Kingdom in the 1950s yet have been the standard for >50 y. Recent analysis of a population-based cohort of healthy children from the United States observed substantial variability in the relation between Tanner stages and timing of peak height velocity (62). This new research suggests that peak height velocity can occur in later stages of puberty. In the study, ∼30% of girls and ∼40% of boys had not attained peak height velocity by the time of menarche and Tanner stage 4 for genitalia, respectively. Considering the potentially limited external validity of the Tanner stages and questions about their accuracy to predict peak height velocity for at least one-third of the population, it is possible that current guidance for maturational adjustments is inadequate.

Furthermore, collecting data on sexual maturation in large-scale surveys and across cultural contexts is challenging (63). Currently, self-assessment and physical examination are the only methods suitable for data collection in field settings. Menarche and spermarche are the only clear and obvious pubertal events. Self-assessment of other signs can be unreliable and subjective, particularly for puberty onset (63, 64). Physical examination requires time, privacy, and trained clinicians who are experienced and comfortable working with adolescents. Even when meeting those conditions, the examination can be perceived as invasive and prone to measurement error (63), and may be unacceptable in some cultures. Other maturation measures, such as assessment of skeletal age using radiographs (e.g., wrist X-rays to assess growth plate closure), may also be unfeasible outside of clinical settings (65). There should be further exploration into the validity and feasibility of methods that measure hormone concentration to estimate timing of peak height velocity (45, 63), strategies examining a smaller subgroup of adolescents to calibrate maturational timing for the broader population, and mathematical modeling to predict the timing and tempo of growth spurts using cross-sectional data (66, 67).

### Racial/ethnic differences in linear growth potential

The MGRS found similarity in linear growth between children <5 y of age in the 6 study sites, supporting the theory and previous empirical findings that population differences in HAZ among young children are predominantly a result of differences in their environment (e.g., nutrition, infectious disease burden) and not inherited traits (35). Without data from a study like MGRS that controls for environmental determinants, it is imprudent to assume that the same is true among older children and adolescents (36, 38), given that large differences exist between global populations in heights of healthy young adults (36, 42). When using the 2007 WHO Growth Reference, the possibility that a given study population has a different growth potential than the reference population should be considered. Several studies from diverse contexts have found differences in the linear growth trajectory of adolescents compared to the 2007 WHO Growth Reference including the Netherlands (68), Peru (69), Argentina (70), Poland (71), and Hong Kong (72). To our knowledge, however, no studies adjusted comparisons for maturational differences between the study and reference populations.

### Limitations of BMI as an indicator of adiposity and thinness and associated health risks among adolescents and across different racial/ethnic populations

Whereas BMI is highly correlated with adiposity for most adult population groups (and with subsequent risk of adult mortality), the association among adolescents is more variable (73, 74). BMI does not distinguish between fat-free mass (e.g., bones, muscles) and fat mass (50, 75), and many different proportions of these can result in the same BMI among adolescents (73). Fat-free and fat mass during adolescence depend on various factors including age, sex, pubertal status, exercise regimen, and race/ethnicity (6, 52, 73, 76–79). Studies indicate that longitudinal increases in BMI during childhood and adolescence are largely attributable to fat-free mass, particularly among adolescent boys (56, 73, 77, 78). The accuracy of BMI as a surrogate measure of adiposity also varies according to total body weight, with BMI performing well among heavy children and adolescents but not among those who are lighter (73, 78). Waist-to-height ratio has been shown to be better than BMI at predicting adiposity in children and adolescents and could be a superior marker of adiposity-related morbidity (80).

Body proportions, build, and fat distribution influence the interpretation of BMI and vary substantially across different racial/ethnic groups (81). Leg length relative to height affects BMI values and potentially distorts classifications of thinness and fatness based on BMI (82–84). Populations that have relatively long legs for their height have lower BMI values which could lead to underestimates of overweight and obesity, and overestimates of thinness (the converse is found for greater BMI associated with relatively shorter leg length). Significant variation in sitting height ratio is found across ethnic groups (30). Slenderness of limbs measured with wrist and knee width, and slenderness of the trunk measured with pelvic breadth and shoulder breadth, also affect interpretation of BMI (85). Populations with more slender builds tend to have a higher percentage of body fat for the same BMI as those with less slender builds (85–87); BMI has been found to systematically underestimate adiposity in South Asian children (88). When using the NCHS/WHO Growth Reference (89) and 2007 WHO Growth Reference (72), a high prevalence of thinness unlikely reflects the true levels of acute malnutrition that have been found in some samples of adolescents from relatively healthy and high-socioeconomic-status Asian populations. The apparent overestimation of thinness is likely due to differences in body build between the study and reference populations. Body fat distribution also varies across populations. For example, South Asians have a more centralized distribution of body fat which coincides with increased risk of diabetes, hypertension, and cardiovascular disease compared to Europeans at the same BMI values (86, 90, 91).

### SDs of height and BMI vary with age and maturation

**FIGURE 3:**
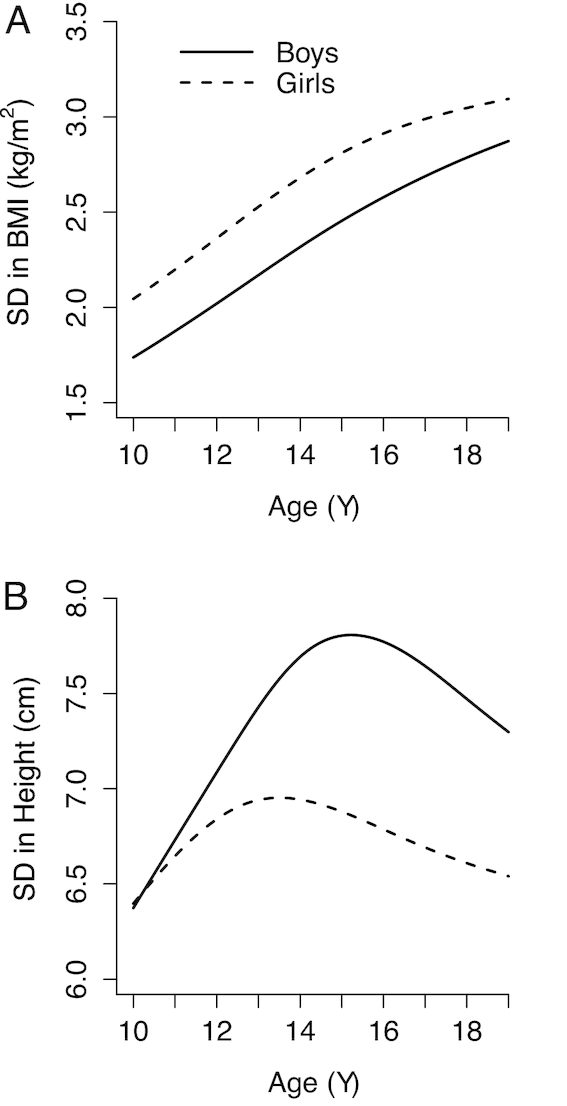
SDs of BMI (A) and height (B), from the 2007 WHO Growth Reference by age and sex (24). Data were fitted using cubic smoothing splines.

**Figure 3** plots the SDs of height and BMI from 10 to 19 y for boys and girls using the 2007 WHO Growth Reference data (24). Height SD increases rapidly from 10 y, peaking at 15 y for boys and 13.5 y for girls before declining steadily; the increase reflects variation in biological maturation of the reference population due to genetic and environmental factors as well as divergence in individual child growth trajectories. The BMI SD continuously increases with age from 10 to 19 y for both sexes. The calculations of HAZ and BMIZ use the SD of the growth reference as the denominator; as a result, for the same difference between the observed value and reference median, the absolute value of the *z* score decreases as SD increases. Accordingly, recent studies have shown that HAZ increases as children and adolescents age (until reaching peak height velocity), and prevalence of stunting decreases, even when height deficits increase (92–95).

The mean of the distribution of height SD of the NCHS/WHO reference population for girls aged 10–14 y is 6.8 cm and for girls 15–19 y 6.7 cm; the mean for boys 10–14 y is 7.1 cm and for boys 15–19 y 7.6 cm (Figure 3). Therefore, there is little impact of differing SDs between younger and older adolescent girls on prevalence estimates for height and only a small impact for boys—older adolescent boys will have an artificially lower prevalence of stunting than younger boys, but the difference will be small. In contrast, for BMI, SD increases with increasing age for both boys and girls (Figure 3). The mean of the distribution of BMI SD for girls aged 10–14 y is 2.36 and for girls 15–19 y is 2.93; the mean for boys 10–14 y is 2.02 and for boys 15–19 y 2.63. For both boys and girls, these large increases in SD with age indicate that a given absolute difference in BMI from the reference will result in lower prevalence estimates of thinness, overweight and obesity at older compared with younger ages.

Sex differences in height and BMI SD between adolescent boys and girls influence prevalence estimates of stunting, thinness, overweight, and obesity based on the 2007 WHO Growth Reference. The mean height SD of the NCHS/WHO reference population is 6.8 cm for girls and 7.4 cm for boys. Therefore, for the same absolute difference in height, girls will have a higher prevalence of stunting than boys. The mean BMI SD for girls is 2.7 and for boys is 2.4. Therefore, for the same absolute difference in BMI, girls will have a lower prevalence of thinness, overweight, and obesity than boys. Separate analysis of the 2011–2012 Bangladesh Integrated Household Survey data found the prevalence of thinness (BMIZ < −2 SD from the 2007 WHO Growth Reference) in adolescents aged 10–20 y was 22% in boys compared to 17% in girls (*P* < 0.05) (96). The difference favoring girls, however, could be a result of sex differences in BMI SD. There are several plausible reasons for why boys may experience a higher burden of thinness than girls, such as a higher level of physical labor and energy requirements among boys. A lower prevalence of thinness among girls than among boys in Bangladesh is unexpected, however, based on reports of gender discrimination in household food distribution (97).

### Relation between cutoffs and risk of adverse outcomes

Despite the limitations discussed thus far in this article, adolescent stunting, thinness, and obesity have been associated with past, present, adult, and intergenerational outcomes. Variation in adolescent HAZ largely reflects variation in growth during childhood, and HAZ is positively associated with school attendance (98, 99) and non-cognitive markers of self-efficacy, self-esteem, and educational aspirations (100), and negatively associated with cognitive performance (98, 101–103) and school performance (98, 101, 103, 104). The evidence of the association between maternal stunted height and negative birth outcomes such as child mortality, stunting, and underweight is well established (105–108). Adolescent thinness is negatively associated with school performance (104, 109). In addition, low maternal BMI in early pregnancy increases the risk of infants being small for gestational age (110), and high maternal BMI before pregnancy is associated with stillbirth, infant mortality, and cerebral palsy in offspring (111, 112). Childhood and adolescent obesity is associated with increased risk of numerous adverse health measures, such as hypertension, insulin resistance, metabolic syndrome, atherosclerosis, and nonalcoholic fatty liver disease (113–115). Although there is little evidence that obesity during childhood and adolescence is an independent risk factor for metabolic disease in adulthood (116, 117), obesity during childhood and adolescence is strongly associated with adult obesity (116–118).

Adolescent anthropometric indicators are clearly useful in determining the risk of various adverse outcomes. The best cutoffs, however, are likely to vary considerably depending on an indicator's use, as described by the WHO Expert Committee on Physical Status (30) and again by Pelletier (32). For example, the best-performing BMIZ cutoff to identify adolescents at present risk of adverse health outcomes may be different from that to identify future risk. Moreover, the same indicators reflect different types of health risks and have different implications depending on age and sex. Ideally, the basis for defining cutoffs should be health and functional consequences associated with deviations in an anthropometric indicator (32). Current adolescent anthropometric cutoffs, however, are not designed to select individuals or populations with specific risks (37). SDs from the reference population median are used to classify individuals who are unusually low, usual, or unusually high based on statistical probabilities (37, 50). Although the 2007 WHO Growth Reference mean, median, and SD provide metrics to assess if there is displacement or distortion of the study population's anthropometric curve compared to the reference data, they provide an inadequate estimate of who is likely to suffer adverse outcomes and what those outcomes are (30).

Perumal et al. (119) explained the misuse of the stunting cutoff (HAZ < −2) to classify undernutrition of children <5 y and to estimate the proportion of a population at risk of adverse outcomes. Many of the commentary's arguments apply to adolescent anthropometric indicators. Like child stunting, the relation between adolescent anthropometric indicators and functional impairment, clinical signs of deficiency, or adverse health outcomes is graduated and seemingly without notable inflection points. There is no single cutoff that distinguishes a well-nourished population from a malnourished one. Consequently, the prevalence of children and adolescents affected by malnutrition may be underestimated when using a single cutoff if the entire distribution of a population is shifted down or up.

Using the same *z* score cutoffs across different anthropometric indicators can be misleading because they represent very different magnitudes of nutrition problems depending on the indicator. For example, among children <5 y old, a stunting prevalence of <20% (based on a HAZ < −2) is considered low, whereas the same prevalence for a weight-for-height *z* score <−2 is high and likely reflects severe food insecurity (30). This point is usually understandable among nutritionists, but less so by nontechnical audiences. For example, the much higher prevalence of adolescent stunting than thinness shown in Figure 1 may falsely give the appearance that stunting is a more prominent public health issue in Bangladeshi adolescent girls than thinness. Thus, presentation of such statistics to policy makers requires substantial explanation and technical support to ensure appropriate prioritization.

### Indicator cutoffs are misaligned across the lifespan

**Table 1** summarizes cutoffs for anthropometric indicators used by WHO across the lifespan. The 1977 NCHS/WHO Growth Reference data were merged with data from the 2006 WHO Child Growth Standard using sophisticated statistical methods to smooth the transition between the 2006 WHO Child Growth Standard for children <5 y and the 2007 WHO Growth Reference for children and adolescents 5–19 y. The cutoffs for stunting and underweight (BMIZ) for children under and over 5 y old are closely aligned (37). WHO recommends a more conservative BMIZ overweight cutoff of +2 for children <5 y old, however, so there is a jump in prevalence at age 5 y when the cutoff is decreased to +1 (120).

**TABLE 1:** Comparison of WHO indicators across the lifespan^1^

Indicator	Cutoff	Corresponding value at 19 y
Children <5 y		
Stunting	HAZ < −2	—
Thinness	BMIZ, WHZ, or WLZ < −2	—
Overweight	BMIZ, WHZ, or WLZ > +2	—
Obesity	BMIZ, WHZ, or WLZ > +3	—
Girls 5–19 y		
Stunting	HAZ < −2	<150.1 cm
Thinness	BMIZ < −2	BMI < 16.5
Overweight	BMIZ > +1	BMI > 25.0
Obesity	BMIZ > +2	BMI > 29.7
Boys 5–19 y		
Stunting	HAZ < −2	<161.9 cm
Thinness	BMIZ < −2	BMI < 17.6
Overweight	BMIZ > +1	BMI > 25.4
Obesity	BMIZ > +2	BMI > 29.7
Adults ≥20 y		
Stunting (women)^2^	Height < 145.0 cm	—
Thinness	BMI < 18.5	—
Overweight	BMI ≥ 25.0	—
Obesity	BMI ≥ 30.0	—

^1^Indicators for children <5 y are based on the 2006 WHO Child Growth Standard (29); indicators for children and adolescents 5–19 y are based on the 2007 WHO Growth Reference (24); and indicators for adults ≥20 y are based on the recommendations by the 1995 WHO Expert Committee (30). BMIZ, BMI (in kg/m^2^)-for-age *z* score; HAZ, height-for-age *z* score; WHZ, weight-for-height *z* score; WLZ, weight-for-length *z* score.

^2^There is no commonly used cutoff for stunting among adult men. Cutoff based on Reference (121).

The larger problem is going from adolescent to adult references. For girls aged 19 y, the 2007 WHO Growth Reference stunting cutoff for children and adolescents aged 5–19 y (HAZ < −2) corresponds with an adult height of <150.1 cm. The cutoff for stunted adult height among women is 145.0 cm and was chosen because it is commonly reported and represents increased obstetric risk (121). The 145-cm cutoff is closer to the 2007 WHO cutoff of severe stunting (HAZ < −3) for adolescent girls aged 19 y (<143.5 cm). This mismatch reveals the unclear meaning of adolescent stunting (i.e., which health risks does it represent?) and causes prevalence of stunting/stunted height to drop substantially from Bangladeshi girls aged 15–18 y (32%; based on HAZ < −2) to women aged 19–22 y (13%; based on height <145 cm) (Figure 1). The 2007 WHO Growth Reference BMIZ values at 19 y closely align with adult BMI cutoffs for overweight and obesity but not for thinness. The BMI values for both sexes at a BMIZ <−2 (17.6 for boys and 16.5 for girls) are considerably lower than the adult thinness cutoff of 18.5 (Table 1 and Figure 2) (30). This is the primary reason we see such a large jump in prevalence of thinness when going from Bangladeshi girls aged 15–18 y (10%; based on BMIZ < −2) to women aged 19–22 y (26%; based on BMI < 18.5) (Figure 1).

Another challenge is identifying at which age to start using adult cutoffs for anthropometric indicators. Young men 20–24 y old can continue to gain height, weight, and muscle mass (4, 6). Moreover, when looking at the rate at which BMI SD changes with age in the 2007 WHO Growth Reference for children and adolescents 5–19 y old, the SD is still increasing at ages 18 and 19 y, especially for boys (Figure 3). As already discussed, growth for many adolescents is still increasing substantially in late adolescence, and therefore using a fixed adult cutoff is inappropriate. The 2007 WHO Growth Reference sample for children and adolescents 5–19 y old ends at age 19 y and 0 mo, 1 y short of when the WHO adult BMI cutoffs were intended to begin (30). Although there are other potential reasons for the considerable increase in thinness and decrease in stunting between girls aged 15–18 y and women aged 19–22 y in Bangladesh, the primary reason is the misalignment of cutoffs transitioning from adolescence to adulthood.

## Conclusion

Improving nutritional status during adolescence is an opportunity to improve the lives of this generation and the next. Estimating the magnitude and distribution of malnutrition at a population level is fundamental to identifying priorities, designing and targeting interventions, and measuring progress over time. For adolescents we usually depend on survey data and the 2007 WHO Growth Reference to do so, with considerable limitations as highlighted here. Young children from diverse racial/ethnic groups grow similarly during the first 5 y of life when their physiological needs are met and environments support healthy development (35, 122). Evidence suggests that this may not be the case as children mature through adolescence as body build and growth potential vary. Nor is chronological age a good proxy indicator of maturation. Thus, determining the true prevalence of undernutrition, overweight, and obesity among adolescents is complicated by racial/ethnic variation across populations in timing of the adolescent growth spurt, growth potential, and body build. Estimating the true burden of disease from malnutrition in adolescence is further complicated by the inherent limitations of BMI as an indicator of thinness and adiposity, and the lack of cutoffs for nutritional status during adolescence that are established based on current or future health risks.

We encourage critical interpretation of HAZ and BMIZ indicators and distributions by age when using the 2007 WHO Growth Reference. Reanalysis of SFSNB data was beyond the scope of this article, and we do not know the magnitude of the shift in prevalence estimates which would result from alignment of maturation and chronological age between the Bangladesh SFSNB survey sample and the reference population. We expect for younger adolescent girls, however, that the prevalence of stunting and thinness should be higher and overweight and obesity lower. Substantially larger BMI SDs in older Bangladeshi adolescent girls likely cause the prevalence of thinness and overweight and obesity to be underestimated relative to younger girls. Assessing the slope of the mean and SD of height-for-age difference, HAZ, BMI-for-age difference, and BMIZ would also elucidate the extent to which prevalence estimates reflect true changes in nutritional status with age and between sexes.

Many of the challenges identified here and the need for further development of indicators and validated cutoffs cannot be easily addressed, and approaches are needed that complement anthropometric indicators in assessing nutritional status during adolescence. Examining what is happening before and after adolescence, when interpretation of anthropometry is more straightforward, and trends over time can provide further evidence of nutritional risk and guide the need for and potential of interventions to address that risk. Prenatal, infant, and early childhood experiences cumulatively affect adolescent development (4) and adolescents are subject to the same secular changes affecting nutritional status of adults (41). Bangladesh has achieved one of the fastest prolonged reductions in stunting among children <5 y of age, with a decrease from 55% in 1996–1997 to 36% in 2014 (123). Among women aged 15–49 y, thinness (BMI < 18.5) declined from 34% in 2004 to 19% in 2014 (124). At the same time, the proportion of overweight women (BMI ≥ 25) has increased from 3% to 24% (124). The SFNSB data and wider population trends support continuation of programs and policies to address the burden of undernutrition while instituting new initiatives to ameliorate an unhealthy nutritional transition in the country. Quantifying the risk of malnutrition during adolescence is critical to informing such a comprehensive strategy.

Proximate and distal determinants of malnutrition can provide proxy indicators of nutritional risk in adolescents, and at the same time provide information on valuable potential entry points for intervention, particularly those measuring dietary intake. Dietary quality plays a key role in multiple forms of malnutrition; 6 of the 11 risk factors driving the global burden of disease are diet-related (125). Whereas there is growing global evidence for adult dietary patterns (126), there is limited evidence on adolescent diets. Knowing where diets are falling short of supporting health and wellbeing, and why, is instrumental to designing nutrition interventions for adolescents. We need standardized indicators validated across contexts to assess dietary quality among adolescents and more explorative studies on determinants of adolescent dietary intake.

Building on the UNICEF conceptual framework of the causes of malnutrition and death among children (127), the WHO recently published a framework of interventions and determinants of adolescent nutrition (17). The most immediate determinants are access to a nutritious diet, positive health behaviors (including food choice), and access to essential health services; underlying determinants include a myriad of factors related to food systems, health care, and water and sanitation infrastructure and services. The WHO conceptual framework helps to identify a broad range of determinants based on current evidence but does not allow for an understanding of the causal paths between individual determinants or provide sufficient insight into which interventions can best address these paths. To know what interventions are necessary in a given context, we need more studies which deepen knowledge of impact paths such as was recently completed by Leroy et al. (96) on the determinants of adolescent nutrition in Bangladesh. Leroy et al.’s analysis indicated that education and empowerment of the female household head are not sufficient to improve the nutritional status of adolescents, suggesting that resources may be too constrained. They concluded that improving dietary intake and nutritional status requires policies and programs that increase household and adolescent access to diverse diets and simultaneously address gender social norms with respect to adolescent nutrition.

In conclusion, we encourage critical thinking in the interpretation of prevalence estimates for anthropometric indicators of nutritional status during adolescence and call for the systematic collection and use of information beyond anthropometry to be given weight in policy and program decision-making. Although reiterating limitations, we are not advocating for the rejection of the 2007 WHO Growth Reference. The global nutrition community faces the same challenges, if not more because of the obesity epidemic, as the expert group responsible for the development of the 2007 WHO Growth Reference who sought to develop a single international growth reference that described universal growth patterns of adolescents. We advocate that nutrition researchers prioritize exploration of better methods to predict peak height velocity which can be applied to cross-sectional surveys, for the development of standardized indicators to measure dietary quality among adolescents, and for studies that will illuminate causal paths so that we can effectively improve adolescent dietary intake and nutritional status.

## Notes

Perspective articles allow authors to take a position on a topic of current major importance or controversy in the field of nutrition. As such, these articles could include statements based on author opinions or point of view. Opinions expressed in Perspective articles are those of the author and are not attributable to the funder(s) or the sponsor(s) or the publisher, Editor, or Editorial Board of *Advances in Nutrition*. Individuals with different positions on the topic of a Perspective are invited to submit their comments in the form of a Perspectives article or in a Letter to the Editor.

Supported by the Ministry of Foreign Affairs of the Netherlands.

Author disclosures: AT, TB, LMN, and EAF, no conflicts of interest.

Abbreviations used: BMIZ, BMI-for-age *z* score; HAZ, height-for-age *z* score; IOTF, International Obesity Task Force; MGRS, Multicentre Growth Reference Study; NCHS, National Center for Health Statistics; SFSNB, State of Food Security and Nutrition in Bangladesh.
